# Enhanced Real-Time Detector for Industrial Vision-Based Corn Impurity Detection

**DOI:** 10.3390/foods15061065

**Published:** 2026-03-18

**Authors:** Xiao Zhang, Yuhang Bian, Xiangdong Li, Haoze Yu, Dong Li, Min Wu

**Affiliations:** 1 Beijing Advanced Innovation Center for Food Nutrition and Human Health, College of Engineering, China Agricultural University, 17 Qinghua Donglu, P.O. Box 50, Beijing 100083, China; b20223070612@cau.edu.cn (X.Z.); byh@cau.edu.cn (Y.B.); s20253071783@cau.edu.cn (X.L.); minwu@cau.edu.cn (M.W.); 2 School of Computer and Artificial Intelligence, Beijing Technology and Business University, Beijing 100048, China; yuhz@btbu.edu.cn

**Keywords:** corn cleaning, RT-DETR, grain quality

## Abstract

The effective cleaning of corn prior to storage is crucial for ensuring grain quality and safety. Traditional Convolutional Neural Network (CNN)-based detection methods often struggle to maintain accuracy in scenarios with dense occlusions. Furthermore, limitations in image quality and feature representation hinder their generalization to diverse impurity types. To address these challenges, this paper proposes an enhanced real-time detector transformer model named RT-DETR-CD (Real-Time Detector Transformer with Convolution and Dynamic Upsampling) for corn impurity detection based on industrial vision. This approach integrates Receptive Field Attention Convolutions (RFAConv) to enhance sensitivity to local texture details and employs the dynamic upsampling operator DySample to restore high-frequency edge information. Additionally, a novel Inner-Shape-IoU loss function is introduced to accelerate bounding box regression for objects with varying aspect ratios. Images were captured using FLIR industrial cameras under controllable annular LED illumination. Experiments on a self-built dataset demonstrate that the proposed model achieves a 4.7% improvement in mean average precision (mAP) and operates at 68 frames per second (FPS), outperforming the original RT-DETR model in both accuracy and speed. This work provides a practical solution for real-time, high-precision impurity detection on grain processing lines.

## 1. Introduction

As a globally significant food crop and industrial raw material, corn’s post-harvest grain quality directly impacts storage safety and the quality of processed products. Should these impurities not be effectively removed, there is a risk of elevated temperatures and mould growth in the grain piles during storage. Furthermore, there is also a risk of damage to subsequent processing equipment. At present, the predominant corn cleaning methods in industrial production principally employ mechanical and physical approaches, including air cleaning, screening, and density separation [1,2]. While these methodologies are technically mature and capable of handling large volumes, they primarily separate materials based on physical characteristics such as size, density, and suspension velocity. Consequently, these devices exhibit substandard separation efficiency for impurities such as stones and moldy kernels that have densities similar to corn kernels or share analogous shapes [1,3]. With the advancement of optoelectronic technology, color sorters based on color characteristics have been widely adopted. These machines utilize RGB sensors to reject off-color particles through threshold segmentation [4,5,6]. However, in the complex realities of industrial online inspection environments, relying solely on color characteristics often proves insufficient to distinguish impurities whose hues closely resemble those of normal corn [7]. Furthermore, traditional color-sorting algorithms exhibit poor robustness against dust interference and lighting variations, making them inadequate for meeting modern agriculture’s demand for high-precision, intelligent detection [8,9].

In recent years, innovative solutions have emerged through the integration of agricultural product quality inspection with computer vision and deep learning technologies. Early studies predominantly employed Support Vector Machines (SVMs) or backpropagation neural networks, utilizing manually designed texture and shape features for classification. However, such approaches exhibit limited generalization capabilities [10,11,12]. With the rise of Convolutional Neural Networks (CNNs), two-stage algorithms represented by Faster Region-based CNN (Faster R-CNN) and single-stage algorithms represented by the You Only Look Once (YOLO) series and Single Shot MultiBox Detector (SSD) have achieved remarkable results in the field of agricultural object detection [13,14,15]. Extensive research indicates that CNN-based models significantly outperform traditional image processing methods in terms of accuracy for corn ear counting, disease identification, and impurity detection tasks [16,17,18]. Nevertheless, contemporary deep learning models continue to encounter difficulties in industrial-grade online corn impurity detection. The performance of these models is highly dependent on image quality. Factors such as low resolution, motion blur, uneven lighting, and sensor noise can significantly degrade feature extraction effectiveness and lead to increased misclassification rates, particularly for small particles or transparent impurities. In industrial scenarios involving conveyor belts, corn kernels and impurities typically appear densely packed and in overlapping configurations. Convolutional Neural Networks primarily focus on local features and, constrained by the size of their receptive fields, exhibit limited effectiveness when handling severely occluded elements like elongated husks or long-range dependencies [19,20]. Furthermore, in pursuit of high-speed detection, many studies favor lightweight models such as YOLOv5s and YOLOv8n. However, this often comes at the cost of reduced detection accuracy for minute impurities, leading to increased false positive rates and even failing to meet industrial requirements [21,22]. Simultaneously, the existing large models feature substantial parameter counts and high computational complexity, making deployment challenging on computationally constrained industrial embedded edge devices and limiting their real-time application on production lines [23].

Researchers implemented multifaceted improvements to the algorithmic architecture to address the challenge of balancing model accuracy with computational speed. Common strategies include incorporating attention mechanisms such as Convolutional Block Attention Module (CBAM), Squeeze-and-Excitation (SE), and Efficient Channel Attention (ECA) to enhance the model’s focus on key features of clutter, or adopting feature pyramids (FPN) and variants like Path Aggregation Network (PANet) and Bidirectional Feature Pyramid Network (BiFPN) to enhance small object detection performance and improve multi-scale feature fusion capabilities [24,25,26,27]. Recently, the Detection Transformer (DETR) has demonstrated superior potential compared to traditional CNNs in handling dense occlusions and complex backgrounds, owing to its unique self-attention mechanism that effectively captures global contextual information within images [28]. However, the original DETR model suffers from slow training convergence and high computational demands during inference, making it difficult to meet the real-time requirements of industrial online detection [29]. Baidu’s Real-Time Detection Transformer (RT-DETR) successfully achieves real-time detection speeds surpassing the YOLO series [30] by employing an efficient hybrid encoder and an uncertainty-minimizing query selection mechanism, while preserving the Transformer’s global perception advantages. While RT-DETR has been shown to demonstrate excellent performance on a variety of datasets, its direct application to corn impurity detection necessitates targeted optimization to address the characteristics inherent to agricultural scenarios, such as the diverse shapes of impurities and complex backgrounds [31,32,33]. To address these challenges, this paper proposes RT-DETR-CD, an enhanced version of the RT-DETR model for corn impurity detection. Its main contributions are threefold:

(1) Introducing receptive field attention convolutions (RFAConv) into the main model enhances sensitivity to local texture details, improving feature extraction capabilities for small irregular impurities.
(2) Replacing the conventional bilinear upscaling commonly used in the neck region with the dynamic upscaling operator DySample, which preserves high-frequency edge information while improving detection performance for slender and transparent objects.
(3) An Inner-Shape-IoU loss function is proposed to accelerate bounding box regression and improve localization accuracy for objects with varying aspect ratios. Experiments on a custom dataset demonstrate that this model achieves enhanced accuracy and real-time performance compared to mainstream detectors.

## 2. Materials and Methods

Samples of Wan Nuo 2000 corn with a moisture content of approximately 25% were produced at the Shangzhuang Experimental Station of China Agricultural University and stored in a refrigerator. The experiment utilizes an industrial camera (Model: BFS-U3–51S5C-C, manufactured by FLIR, Inc., Wilsonville, OR, USA) combined with a ring LED light (RI15045-W, produced by OPT-Machine, Dongguan, China) to simulate actual production scenarios.

### 2.1. Data Collection, Processing, and Enhancement

The images were captured using Spinnaker 2.6.0.160 software (FLIR Systems, Inc., Wilsonville, OR, USA) and an industrial camera, yielding 750 raw corn images containing impurities. These impurities included substandard corn kernels, corn husks, corn stalks, corn cobs, weeds, gravel, and glass fragments. The corn kernels and impurities in the images were annotated using the LabelImg annotation tool (v1.8.1). Images were randomly divided into training, validation, and test sets, maintaining a ratio of 4:1:1 [34]. All images were annotated by two trained annotators using LabelImg, and all labels were independently reviewed by a third expert to ensure consistency and accuracy. Any discrepancies were resolved through discussion. Table 1 shows the types and quantities of labeled samples.

**Figure 1:**
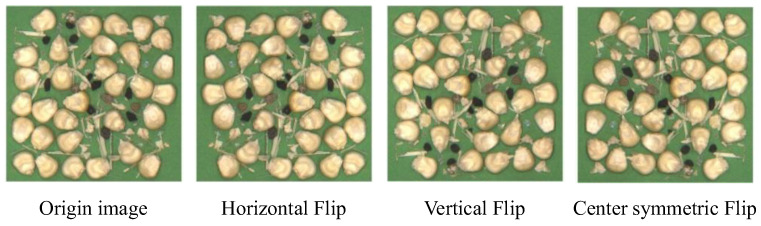
Image augmentation results.

This study employed three data augmentation methods: horizontal flipping, vertical flipping, and center-symmetric flipping. The augmented results are shown in Figure 1. To enhance the model’s generalization capability and prevent overfitting, data augmentation techniques were applied to both the training and validation sets. The test set exclusively contained original, unaugmented images to ensure the impartiality of evaluation results. Following these procedures, a dataset comprising 2100 training images, 520 validation images, and 100 test images was obtained.

### 2.2. Experimental Environment and Parameter Settings

The computational core of this experiment employs high-performance Graphics Processing Units (GPUs), leveraging their parallel computing capabilities to accelerate model training and inference processes. The specific hardware and software configurations are detailed in Table 2.

The input image size setting of 640 × 640 aims to balance the resolution required for detecting minute impurities with the computational constraints of industrial real-time deployment. Experience indicates that an initial learning rate of 0.01 combined with cosine annealing enables stable convergence of the Stochastic Gradient Descent (SGD) optimizer. The batch size is set to 32. By monitoring the validation set loss, the total number of training iterations (epochs) should be set to 300 rounds, with the first 50 rounds designated as a warm-up phase to ensure the model fully converges. The momentum factor is set to 0.937, and the weight decay is set to 0.0005.

### 2.3. Backbone Network Optimization: Embedded Receptive Field Attention Convolution (RFAConv)

**Figure 2:**
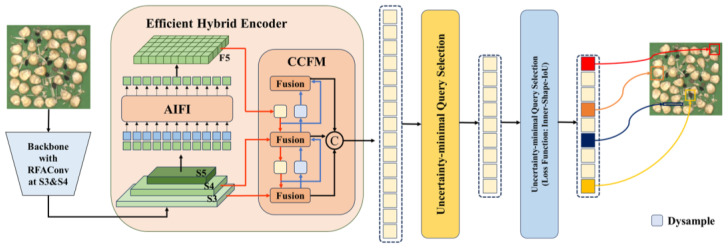
Schematic Diagram of Original RT-DETR Image and RFAConv Module Insertion Position.

In order to address the challenges presented by corn impurity detection within industrial conveyor belt environments, such as high texture similarity, blurred impurity edges, and dense small targets, this paper improves upon the original RT-DETR model and proposes an enhanced real-time detection algorithm [35]. Specifically, we introduce the RFAConv module into the original RT-DETR backbone network and incorporate the DySample dynamic upsampling operator into the neck network. Additionally, we introduce an internal shape intersection loss function into the new model. The backbone network in the original RT-DETR model typically employs ResNet or HGNetv2, where the core convolutional layers share parameters across the entire feature map. Whilst this parameter-sharing mechanism reduces computational complexity, it also disregards the differential information present at different spatial locations within the feature map. Consequently, the model demonstrates an inadequate level of sensitivity to subtle local texture variations. In order to address the aforementioned issues, the present paper replaces the standard 3 × 3 convolutions in the deep feature extraction stages of Stage 3 and Stage 4 within the backbone network with the RFAConv module, as shown in Figure 2.

**Figure 3:**
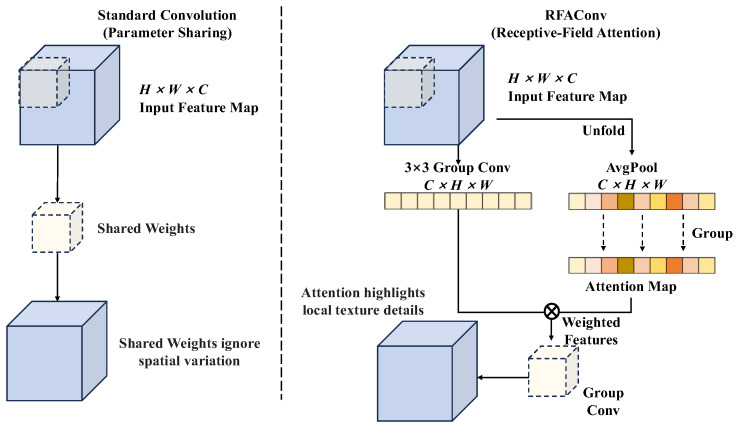
Schematic Diagram of the RFAConv Model.

RFAConv reengineers the convolutional operation pipeline from the perspective of receptive field spatial features [36]. Traditional convolutional operations extract features through sliding windows, whereas RFAConv first employs an unfold operation to extract spatial features within the receptive field. Subsequently, it utilizes an attention mechanism to assign independent weights to each feature point within the receptive field. Finally, feature mapping is achieved through grouped convolutions, as illustrated in Figure 3. Specifically, RFAConv no longer employs the same parameters at all positions to extract features like standard convolutions. Instead, it dynamically adjusts the weights of the convolutional kernel parameters based on the specific content within the receptive field of the input feature map. This enables the network to focus more on the pixel regions within the receptive field that are most discriminative for classification. Standard convolutions may smooth out subtle textural differences in fine features, whereas RFAConv, through its attention mechanism, can acutely capture pixel variations in coarse surface textures within the local receptive field.

### 2.4. Neck Network Reconstruction: DySample Dynamic Upsampling

**Figure 4:**
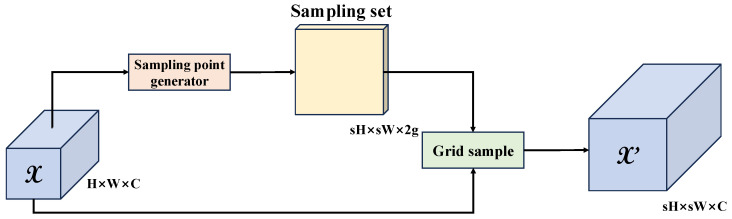
Schematic Diagram of Dynamic Upsampling Method.

In hybrid encoders for RT-DETR, Feature Pyramid Networks (FPNs) are frequently employed. These networks integrate high-level semantic features with low-level detail features through a top-down path [37]. The original model employed the nn.Upsample operation based on bilinear interpolation during this process, with the scale_factor set to 2. Traditional bilinear interpolation is a static linear transformation whose sampling kernel is fixed. It calculates pixel values solely based on geometric distance, disregarding the semantic information within the image content. This operation is similar to a low-pass filter, which can easily cause the feature map after upsampling to become blurred and lose high-frequency detail information. In order to achieve this objective, the present paper introduces the Dynamic Upsampling operator to reconstruct the neck network [38], as shown in Figure 4. DySample is an ultra-lightweight dynamic upsampling method whose core concept involves a shift from simple interpolation to a “point sampling” perspective. It learns the flow field of input features through a lightweight subnetwork, thereby generating a dynamic sampling grid. Given the input image features X and the sampling grid G generated by the model, the output features Y can be derived using the following formula:

Y = S (X, G)(1)

where S represents the point sampling operation, and G is the content-aware offset generated from the input feature X. DySample achieves adaptive upscaling based on the distribution of feature content without requiring the substantial increase in parameters and computational complexity associated with deconvolution.

### 2.5. Loss Function Upgrade: Inner-Shape-IoU

**Figure 5:**
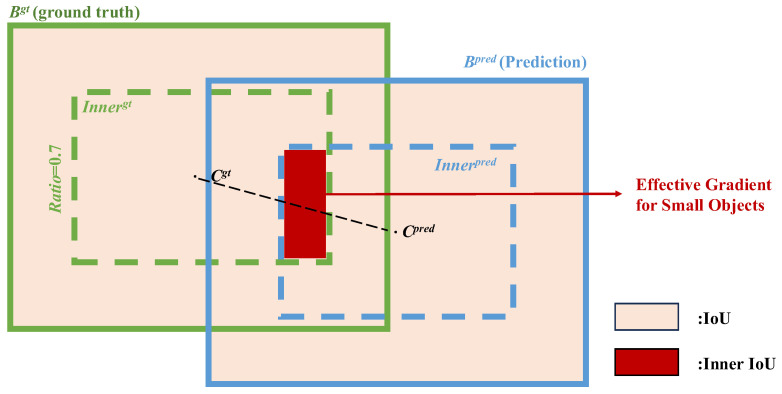
Schematic Diagram of the Inner-Shape-IoU Loss Function.

During the training process of object detection, the bounding box regression loss function directly determines the accuracy of localization. The original RT-DETR employs Generalized Intersection over Union (GIoU) or Complete Intersection over Union (CIoU) as the regression loss [39]. However, when confronted with unstructured agricultural environments, the existing loss functions have been shown to have limitations. In order to address this issue, this paper puts forward the Inner-Shape-IoU loss function. This loss function combines the advantages of both the Shape-IoU and Inner-IoU formulas: while traditional IoU primarily focuses on the area of overlap, Shape-IoU further considers the shape and scale features of the bounding boxes [40,41]. The introduction of aspect ratio as a constraint is pivotal in facilitating the model’s acquisition of the target’s intrinsic geometric configuration. In order to address the challenge of matching difficulties caused by vanishing or unstable gradients in small-target IoU, Inner-IoU introduces an auxiliary scale factor ratio. It calculates IoU by generating smaller auxiliary boxes centered on the Ground Truth (GT) and anchor boxes, as shown in Figure 5.

The improved formula proposed in this paper is defined as follows:

(2) $$L_{Inner\text{-}Shape\text{-}IoU}=1-IoU_{inner}+R_{shape}$$

where *IoU_inner_* is the inner intersection-over-union score calculated based on the auxiliary scale factor ratio:

(3) $$b_{inner_{g}t}=b_{c_{g}t}\pm ratio\cdot \left(b_{gt}-b_{c_{g}t}\right)$$

(4) $$b_{inner_{p}red}=b_{c_{p}red}\pm ratio\cdot \left(b_{pred}-b_{c_{p}red}\right)$$

## 3. Results and Discussion

### 3.1. Comparison Experiment

To validate the comprehensive performance of the improved RT-DETR model, we selected the most widely used models in the field of agricultural engineering: YOLOv5s, YOLOv8n, YOLOv10, along with the original RT-DETR and DETR as controls for this experiment [42,43,44,45]. Evaluation metrics included mean mAP50, number of parameters (Params), and frames per second (FPSs). To ensure fair comparison, all benchmark models were trained from scratch on our dataset under the identical experimental settings shown in Table 2.

As shown in Table 3, the RT-DETRCD model proposed in this paper demonstrates significant advantages in accuracy compared to other models. Compared to the original RT-DETR, the improved model achieves a 3.3% increase in the mAP50, indicating enhanced capability in characterizing corn impurity features. Although the introduction of the RFAConv and DySample modules slightly increased the number of parameters by 0.7 million and FPS from 74 to 68, it remains above the real-time standards typically required for industrial production lines, achieving an effective balance between high precision and real-time performance. Although mAP50 improved by 4.7%, mAP50:95 slightly decreased from 68.2% to 65.2%. These results indicate the model performs well under coarse IoU thresholds but requires further optimization for high-precision localization under stricter IoU standards. Nevertheless, in industrial sorting applications, mAP50 typically better reflects practical performance, as moderate localization errors do not significantly impact downstream processing.

To further investigate the model’s performance across different impurity types, we calculated the AP50 values for each category, as shown in Table 4. Compared to the baseline model, our model achieved significant improvements in mAP for glass shards and corn stalks, increasing by 5.8% and 4.2%, respectively. This demonstrates that the DySample incorporated into our model exhibits a distinct advantage in preserving edge details of transparent and slender objects. For other object categories, mAP values also improved across the board. These results confirm that the DySample module effectively addresses the specific challenges posed by diverse impurity shapes and textures.

**Figure 6:**
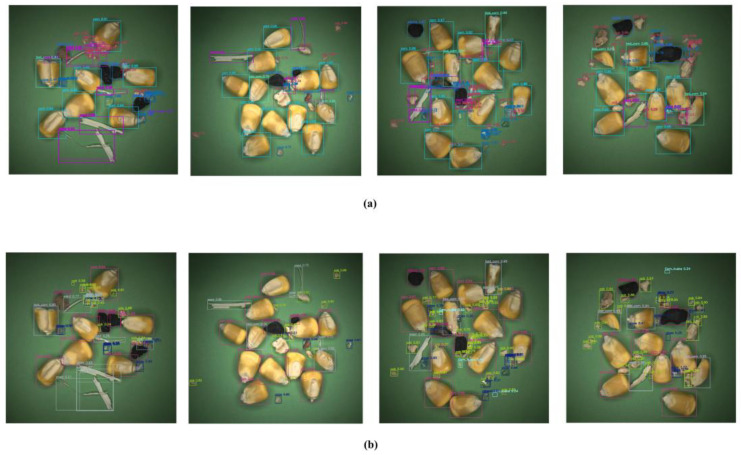
Visualization of inference results: the triangle box represents missed detection. (**a**) RT-DETR. (**b**) RT-DETRCD.

In detection tests targeting the specific category of glass fragments, experimental data indicates that simply replacing the traditional bilinear upsampling method in the neck network with DySample directly increased the recall rate for this category by 5.8%, as shown in Figure 6. In deep feature maps, the edges of transparent glass fragments constitute high-frequency information that is highly susceptible to being smoothed out during traditional interpolation processes. Visual results demonstrate that following the application of DySample, activation values along the glass edges are significantly enhanced, indicating that content-aware sampling successfully preserves sharp geometric edges while preventing their misclassification as background noise.

**Figure 7:**
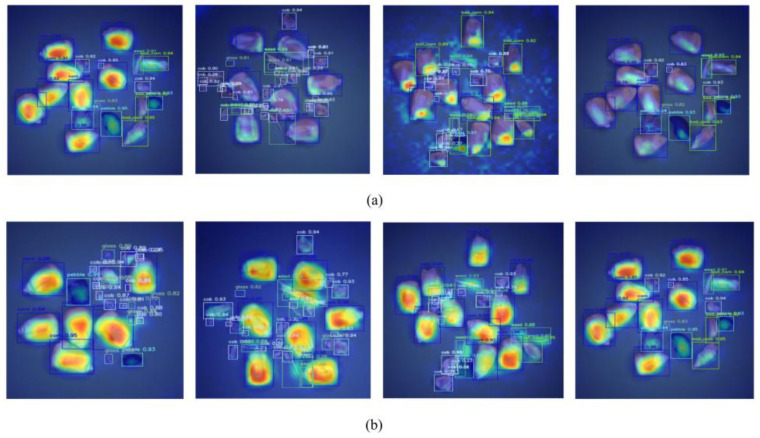
Heatmap: (**a**) RT-DETR. (**b**) RT-DETRCD.

The heatmap visualization results for the improved RT-DETRCD and the original RT-DETR are shown in Figure 7, illustrating the difference in feature learning capabilities between RT-DETRCD and RT-DETR. To more intuitively observe the model’s feature learning capability, the generated heat gradients are confined within the prediction bounding box. Although RT-DETR achieves superior target localization, our model demonstrates more precise feature learning than RT-DETR, effectively focusing on the target object while achieving higher contour fitting accuracy.

### 3.2. Ablation Experiment

The ablation experiments validated the effectiveness of the improvements. These experiments were conducted under identical hardware specifications, software environments, and hyperparameter settings to ensure their validity. The baseline model refers to the original RT-DETR. The results shown in Table 5 indicate that the RFAConv module significantly improves model performance, particularly in achieving a 1.5% increase in mAP50:95. The corn impurity detection task involves numerous irregularly shaped targets. We believe the primary reason for the improved performance of the modified model is that the introduction of the RFAConv module enhances the model’s ability to learn such targets. The introduction of Dysample improves recognition accuracy for dense small objects, increasing the *p*-value by 0.6%. The incorporation of the Inner-Shape-IoU loss function enables the model to better capture the boundary features of objects. This resulted in a 0.7% increase in the R value and a 0.9% increase in the mAP50:95 value. The results of the ablation experiments demonstrate the effectiveness of the RFAConv, Dysample, Inner-Shape-IoU, and RT-DETRCD models.

**Figure 8:**
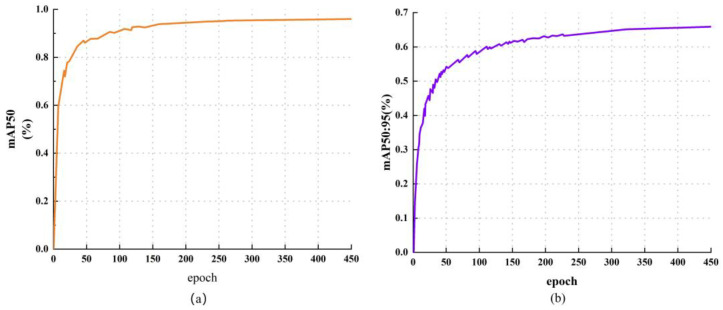
Train process: (**a**) mAP50 vs. epoch; (**b**) mAP50:95 vs. epoch.

The confusion matrix comparison for the two highly confusable targets, dark corn cobs and dark pebbles, is shown in Figure 8. In comparison with the utilisation of CIoU, models employing Inner-Shape-IoU attained approximately 30% faster convergence within the initial 50 epochs. The Inner-IoU mechanism utilises auxiliary small boxes to generate larger gradient backpropagation at low overlap rates, accelerating early weight updates. The experimental findings pertaining to the aspect ratio adaptability of the loss function have demonstrated that this function attains maximum detection accuracy within the High Aspect Ratio category of corn stalks. This verifies the efficacy of the Shape-IoU term in constraining irregular shape regression.

### 3.3. Discussion

This study proposes a novel RT-DETR detector based on the RT-DETR series to enable intelligent corn cleaning operations. Utilizing RFAConv as its backbone network, the model dynamically adjusts the weights of positions within the receptive field, significantly enhancing sensitivity to local texture details and effectively resolving false detection issues caused by metameric impurities. Furthermore, to address edge blurring and small-object feature loss during feature sampling caused by traditional bilinear interpolation, we introduce the content-aware dynamic sampler DySample. This achieves pixel-level point-to-point feature restoration, improving reconstruction accuracy for impurity edges like glass fragments and slender stems by 5.8% and 4.2%, respectively. Simultaneously, we constructed an Inner-Shape-IoU loss function that integrates Shape-IoU’s constraint capability on bounding box shape and aspect ratio with Inner-IoU’s enhanced mechanism for gradient backpropagation of small targets. This approach significantly accelerates model convergence while substantially improving localization accuracy and recall for dense small targets and irregularly shaped impurities. The final model achieves 96.2% mAP50 and 65.2% mAP50:95 detection accuracy on our self-built dataset, running at 68 frames per second. Its overall performance shows significant improvements over mainstream models such as YOLOv5s, YOLOv8n, YOLOv10-S, and the original RT-DETR. Comparative experiments demonstrate that the enhanced precision of this model renders it applicable for agricultural production. This research provides a relatively accurate method for determining impurity content in corn awaiting cleaning, thereby laying the foundation for implementing intelligent cleaning processes.

Although the improved RT-DETR model proposed in this study achieved relatively satisfactory performance in corn impurity detection tasks, certain limitations remain. The current model has 33.5 million parameters and an inference speed of 68 FPS. While it meets real-time detection requirements, there is still room for optimization on computationally constrained embedded edge devices such as Jetson Orin and FPGAs. Subsequent steps may involve applying techniques such as channel pruning, knowledge distillation, and TensorRT inference acceleration to the improved model, further reducing its size and enhancing deployment efficiency. Regarding image-based tasks, this study focuses solely on detection using RGB images. In extreme dusty environments or low-light conditions, pure vision-based methods may face performance degradation risks. Future research may explore integrating multimodal data such as near-infrared, hyperspectral, or depth images to enhance the model’s adaptability to complex industrial environments. Regarding adaptability to detection environments, the data collection for this study was conducted under controlled lighting conditions. Future work will further validate the robustness of this model in complex real-world scenarios involving varying lighting conditions, light direction, object occlusion percentages, and motion blur. Currently, the model is optimized solely for corn impurities. Future research may explore its transferability to detecting impurities in other grains.

## 4. Conclusions

This paper proposes an enhanced real-time detector RT-DETR-CD for corn impurity detection in industrial vision. The model integrates three key innovations: introducing RFAConv in the backbone network to enhance local texture sensitivity, adopting DySample dynamic upsampling in the neck network to preserve high-frequency edge information, and proposing a novel Inner-Shape-IoU loss function to accelerate bounding box regression for objects with varying aspect ratios. Experiments on a self-built corn impurity dataset demonstrate that RT-DETR-CD achieves a mAP50 of 96.2% at an inference rate of 68 FPS, outperforming state-of-the-art detectors such as YOLOv5s, YOLOv8n, YOLOv10, and the original RT-DETR. Ablation studies validate the contributions of each proposed module. The results demonstrate that the proposed method effectively addresses the challenges of detecting small-sized, irregular, and transparent impurities in actual grain processing production lines, providing a practical and high-precision solution for intelligent post-harvest cleaning equipment.

## Figures and Tables

**Table 1:** Types and quantities of samples to be tested.

Samples	Quantities
Corn husk	2100
Corn stalk	1800
Corn cob	1200
Weed	950
Gravel	800
Glass	600
Moldy kernel	1500

**Table 2:** Model running environment configuration details.

Items	Detailed Specifications
CPU	Intel Core i7-14700K
GPU	NVIDIA GeForce RTX 4060Ti (16 GB)
Memory	32 GB
Operating System	Windows 11
Deep learning framework	PyTorch 1.13.1
Programming language	Python 3.8

**Table 3:** Performance comparison of different detection algorithms.

Models	mAP50 (%)	mAP50:95 (%)	Parameters (M)	GFLOPS ^1^	FPS
YOLOv5s	86.2	61.5	7.2	16	115
YOLOv8n	89.4	65.8	3.2	9	142
YOLOv10	90.1	66.5	8.0	22	120
DETR	78.5	52.3	41.3	86	28
RT-DETR (Original)	91.5	68.2	32.8	50	74
Ours	96.2	65.2	33.5	56	68

^1^ GFLOPS: Giga Floating-Point Operations per second.

**Table 4:** Detection performance for different types of materials.

Impurity Category	Baseline mAP50 (%)	Ours mAP50 (%)
Corn husk	92.3	94.5
Corn stalk	88.5	92.7
Corn cob	90.1	91.8
Weed	89.7	91.2
Gravel	91.0	92.3
Glass fragment	84.3	90.1
Moldy kernel	93.5	94.6

**Table 5:** Architectural ablation experiment.

Models	P ^1^ (%)	R ^2^ (%)	mAP50 (%)	mAP50:95 (%)
Base model	94.2	89.5	91.5	68.2
+ DySample	94.8	91.6	94.8	61.4
+ RFAConv	95.1	90.3	94.2	60.2
+ Inner-Shape-IoU	94.5	90.8	93.9	59.6
+ DySample + RFAConv	95.7	92.4	95.4	63.1
Ours	96.3	93.8	96.2	65.2

^1^ P: Precision; ^2^ R: Recall.

## Data Availability

The data presented in this study are available on request from the corresponding author. The complete raw dataset is not publicly available due to confidentiality agreements regarding the algorithms.

## Abbreviations

The following abbreviations are used in this manuscript:

RT-DETR	Real-Time Detection Transformer
RT-DETRCD	RT-DETR with Convolution and Dynamic upsampling
CNN	Convolutional Neural Network
RFAConv	Receptive Field Attention Convolution
DySample	Dynamic Upsampling operator
Inner-Shape-IoU	Inner-Shape Intersection over Union
mAP	mean Average Precision
FPS	Frames Per Second
YOLO	You Only Look Once
SVM	Support Vector Machine
SSD	Single Shot MultiBox Detector
Faster R-CNN	Faster Region-based CNN
CBAM	Convolutional Block Attention Module
SE	Squeeze-and-Excitation
ECA	Efficient Channel Attention
FPN	Feature Pyramid Network
PANet	Path Aggregation Network
BiFPN	Bidirectional Feature Pyramid Network
DETR	Detection Transformer
SGD	Stochastic Gradient Descent
ResNet	Residual Network
GIoU	Generalized Intersection over Union
CIoU	Complete Intersection over Union
IoU	Intersection over Union
GT	Ground Truth
GFLOPS	Giga Floating-Point Operations Per Second
P	Precision
R	Recall
